# Ubiquitin-conjugating enzyme 2C (UBE2C) is a poor prognostic biomarker in invasive breast cancer

**DOI:** 10.1007/s10549-022-06531-5

**Published:** 2022-02-06

**Authors:** Yousif Kariri, Michael S. Toss, Mansour Alsaleem, Khloud A. Elsharawy, Chitra Joseph, Nigel P. Mongan, Andrew R. Green, Emad A. Rakha

**Affiliations:** 1 Academic Unit for Translational Medical Sciences, School of Medicine, University of Nottingham Biodiscovery Institute, University Park, Nottingham, NG7 2RD UK; 2 Department of Clinical Laboratory Science, Faculty of Applied Medical Science, Shaqra University, 33, Shaqra, 11961 Saudi Arabia; 3 Department of Applied Medical Science, Applied Collage in Unazyzah, Qassim University, Qassim, Saudi Arabia; 4 Department of Zoology, Faculty of Science, Damietta University, Damietta, 34517 Egypt; 5 Biodiscovery Institute, Faculty of Medicine and Health Sciences, School of Veterinary Medicine and Science, University of Nottingham, Nottingham, NG7 2RD UK; 6 Department of Pharmacology, Weill Cornell Medicine, New York, NY 10065 USA; 7 Department of Histopathology, Nottingham University Hospital NHS Trust, City Hospital Campus, Hucknall Road, Nottingham, NG5 1PB UK

**Keywords:** UBE2C, Breast cancer, Lymphovascualr invasion, Progression, Prognosis, Outcome

## Abstract

**Background:**

The Ubiquitin-conjugating enzyme 2C (UBE2C) is essential for the ubiquitin–proteasome system and is involved in cancer cell migration and apoptosis. This study aimed to determine the prognostic value of UBE2C in invasive breast cancer (BC).

**Methods:**

*UBE2C* was evaluated using the Molecular Taxonomy of Breast Cancer International Consortium (*n* = 1980), The Cancer Genome Atlas (*n* = 854) and Kaplan–Meier Plotter (*n* = 3951) cohorts. UBE2C protein expression was assessed using immunohistochemistry in the BC cohort (*n* = 619). The correlation between UBE2C, clinicopathological parameters and patient outcome was assessed.

**Results:**

High *UBE2C* mRNA and protein expressions were correlated with features of poor prognosis, including high tumour grade, large size, the presence of lymphovascular invasion, hormone receptor negativity and HER2 positivity. High *UBE2C* mRNA expression showed a negative association with E-cadherin, and a positive association with adhesion molecule N-cadherin, matrix metalloproteinases and cyclin-related genes. There was a positive correlation between high UBE2C protein expression and cell cycle-associated biomarkers, p53, Ki67, EGFR and PI3K. High UBE2C protein expression was an independent predictor of poor outcome (*p* = 0.011, HR = 1.45, 95% CI; 1.10–1.93).

**Conclusion:**

This study indicates that UBE2C is an independent prognostic biomarker in BC. These results warrant further functional validation for UBE2C as a potential therapeutic target in BC.

**Supplementary Information:**

The online version contains supplementary material available at 10.1007/s10549-022-06531-5.

## Introduction

Breast cancer (BC) is a heterogeneous disease comprising several biological subtypes and shows diverse behaviours and responses to therapy [1]. In-depth investigation of the transcriptomic and proteomic expression of the underlying genetic pathways which contribute to both invasion and metastasis can be critical to decipher the complex molecular makeup of BC and refine and improve its clinical management.

The ubiquitination process is an essential protein degradation mechanism that serves to protect cellular integrity by degrading abnormal and short-life proteins. Moreover, it contributes to the cellular processes that induce cell cycle progression, transcription and apoptosis [2]. Ubiquitin-conjugating enzyme 2C (UBE2C) is a participant in the ubiquitin-conjugating enzyme complex, and it also plays an essential role in the ubiquitin–proteasome system, which normally regulates key checkpoints in the cell cycle via targeting the cell cycle regulators [3]. The UBE2C-encoded protein is involved in mitotic cyclin destructions and cell cycle progression; hence, it potentially could participate in cancer development [4]. Previous studies have identified high UBE2C expression in several types of cancer, including head and neck squamous cell carcinoma [5], gastrointestinal [6] and endometrial cancer [7].

Lymphovascular invasion (LVI), which is indicated by the presence of tumour cells within lymphatic vessels, is considered one of the prerequisites for BC metastasis [8–10]. However, the key molecular processes associated with BC-LVI progression remain poorly understood. Hence, further investigations are required to detect both biological and molecular mechanisms underlying LVI. The results of such investigations should prove vital in terms of developing targeted treatment strategies that can help in improving patient outcomes. Although several prior studies have reported that high expression of UBE2C plays a major role in the progression of BC [11–15], but its role in BC-LVI remains unclear. Based on the findings of the aforementioned studies, we hypothesised that UBE2C plays a significant role in BC progression and metastasis. Here, we investigated the expression of UBE2C in BC at both the transcriptomic and proteomic levels to determine its association with various clinicopathological features including LVI, other related genes and patient outcomes using several well-characterised BC cohorts and datasets.

## Material and methods

### Study cohorts

To investigate the prognostic significance of *UBE2C* mRNA expression in BC, gene expression data were obtained from the TNM plot (https://www.tnmplot.com/) and UALCAN (http://ualcan.path.uab.edu/index.html) datasets, which together include 1097 primary, 7 metastatic tumours and 113 normal tissue samples [16, 17]. Likewise, both the Molecular Taxonomy of Breast Cancer International Consortium (METABRIC) (*n* = 1980) [18] and The Cancer Genome Atlas (TCGA) (*n* = 854) [19] datasets were used as discovery cohorts to assess and explore the prognostic value of *UBE2C* expression at the genomic level. To validate the prognostic value of *UBE2C* mRNA expression, the Kaplan Meier (KM) Plotter (*n* = 3951) online dataset (https://kmplot.com/analysis/) [20], was used.

UBE2C protein expression was measured by immunohistochemistry (IHC) in a large BC cohort (*n* = 619) with detailed clinical information comprising patients presented at Nottingham City Hospital, Nottingham, United Kingdom as previously described [21]. For management purposes, Nottingham Prognostic Index (NPI) and Oestrogen Receptor (ER) status were used to classify patients into clinically relevant groups. Patients with a good prognostic NPI score (≤ 3.4) received no adjuvant therapy, whereas patients with poor prognostic NPI score (> 3.4) received endocrine treatment if ER status was positive and received chemotherapy [classical cyclophosphamide, methotrexate and 5-fluorouracil (CMF)] if ER status was negative. None of the patients in this study received neoadjuvant therapy or anti-human epidermal growth factor receptor 2 (HER2) targeted therapy. The clinicopathological features for the cohort series were summarised previously [21, 22].

To investigate the interactions between UBE2C expression and other related biomarkers, previous available data [23–25] have been used. This includes DNA and cell cycle regulator (p53, CDCA5), proliferation marker (Ki67), adhesion molecules (E-cadherin (CDH1) and N-cadherin (CDH2), basal-phenotype (CK5 and CK14 positive), phosphatidylinositol 3-kinase (PI3K) and epidermal growth factor receptor (EGFR).

### UBE2C protein expression evaluation

Prior to IHC staining, the validity of the primary UBE2C antibody (WHO0011065M1, Sigma-Aldrich, Gillingham, UK, 1:300) was checked using immunoblotting. The specificity of the UBE2C was validated in SKBR3 human BC cells (obtained from the American Type Culture Collection, Rockville, MD, USA). The rabbit β-actin antibody (A5441, clone AC-15, Sigma-Aldrich, Gillingham, UK) was used at 1:5000 as a housekeeping protein and showed a band at approximately 42 KDa. A single specific band for the UBE2C protein was detected at the expected molecular weight of ~ 20 KDa after incubation overnight (Supplementary Fig. 1A).

Fourteen full face sections of BC cases, representative of several molecular subtypes and tumour grade, were used to evaluate the distribution of UBE2C expression. Patients’ samples were arrayed into tumour microarrays (TMA) as previously described [26]. Citrate antigen retrieval (pH 6.0) was used, and samples were incubated overnight at 4 °C with UBE2C antibody diluted (1:100). Novolink Max Polymer Detection kit (Leica, Newcastle, UK) was used to express the immunoreactivity of UBE2C [21]. UBE2C-stained slides were scanned using high-resolution digital images (NanoZoomer; Hamamatsu Photonics, Welwyn Garden City, UK) at 20 × magnification and visualised on viewing software (Xplore; Philips, UK) to assess the protein expression level. A semi-quantitative evaluation was used to assess a modified histochemical score (H-score) [27] which is combined with the staining intensity (0–3) multiplied by the proportion of tumour cells (0–100). The staining intensity was categorised into four groups: 3 (strong staining); 2 (moderate staining); 1 (weak staining) and 0 (no staining). The final H-score was obtained by giving a range of 0 to 300. Cores with less than 15% tumour areas and/or with folded tissue were not assessed. The interobserver concordance was checked by doing a blind double scoring for two researchers (YK and SA).

### Statistical analysis

The data analysis was presented using SPSS statistical software (IBM SPSS Statistic, Version 24.0, Chicago, IL, USA). The mRNA and protein expressions were categorised into low and high subgroups according to their median (METABRIC; 9.13, TCGA; 533, protein; 20 H-score) cut-off. Inter-observer agreement in UBE2C IHC scoring was evaluated using intra-class correlation coefficient (ICC). The associations between mRNA expression of *UBE2C* and adhesion molecules, metalloproteinase (*MMPs*), cyclin and cell cycle-related genes were analysed by using Person’s correlation test. The Chi square test was used to study the correlation between UBE2C expression and the other categorical variables in both transcriptomic and proteomic levels. Kaplan–Meier survival test was performed to assess the correlation with patients’ outcome. Cox regression model was used for multivariate analysis. *P* value of < 0.05 was used to detect the statistical significance.

This study followed the reporting recommendations for tumour markers prognostic studies (REMARK) criteria [28].

## Results

### Transcriptomic and genomic expression of *UBE2C*

In both the TNM plotter and ULACAN datasets, high *UBE2C* mRNA expression was identified more in BC when compared with the normal breast tissues (Supplementary Fig. 1B). Among the different molecular subtypes, the expression of *UBE2C* was higher in the HER2-enriched BC and triple negative (TNBC) than in the luminal-A class (Supplementary Fig. 1C; Table 1). High *UBE2C* mRNA expression was significantly associated with the presence of LVI (METABRIC cohort: *p* = 0.002, TCGA cohort: *p* < 0.001) and other factors characteristics of a poor prognosis, including larger tumour size (*p* < 0.001), high tumour grade (*p* < 0.001), ER and progesterone receptor (PR) negativity (*p* < 0.001) and HER2 positivity (*p* < 0.001; Table 1). High *UBE2C* expression was also associated with a high nodal stage in the METABRIC cohort (*p* < 0.001) (Table 1).

**Table 1:** Association of *UBE2C* mRNA expression with clinicopathological characteristics in the Molecular Taxonomy of Breast Cancer International Consortium (METABRIC) (*n* = 1980) and in the Cancer Genome Atlas (TCGA) (*n* = 854) breast cancer series

Parameters	METABRIC cohort			TCGA cohort		
	Low *UBE2C*	High *UBE2C*	*p* value	Low *UBE2C*	High *UBE2C*	*p* value
	*N* (%)	*N* (%)		*N* (%)	*N* (%)	
Tumour size						
≤ 2.0 cm	492 (57)	367 (43)	** < 0.001**	145 (61)	49 (39)	** < 0.001**
> 2.0 cm	492 (45)	**609 (55)**		286 (46)	**332 (56)**	
Lymph Node status						
Negative	566 (55)	469 (45)	** < 0.001**	219 (51)	207 (49)	0.471
Positive	421 (45)	**517 (55)**		207 (49)	216 (51)	
Histological grade						
Grade 1 and 2	677 (72)	263 (28)	** < 0.001**	333 (72)	131 (28)	** < 0.001**
Grade 3	250 (26)	**702 (74)**		71 (20)	**281 (80)**	
Tumour histological subtypes						
Ductal NST	684 (44)	**860 (56)**	** < 0.001**	298 (51)	300 (49)	0.447
Lobular	17 (53)	15 (47)		93 (52)	84 (48)	
Medullary like	163 (80)	40 (20)		15 (53)	13 (47)	
Special type	103 (70)	44 (30)		14 (52)	13 (48)	
Lymphovascular invasion (LVI)						
Negative	492 (53)	438 (47)	**0.002**	315 (56)	244 (44)	** < 0.001**
Positive	286 (45)	**349 (55)**		113 (38)	**182 (62)**	
Oestrogen receptor (ER)						
Negative	98 (21)	**376 (79)**	** < 0.001**	24 (13)	**161 (87)**	** < 0.001**
Positive	892(59)	614 (41)		391 (61)	248 (39)	
Progesterone receptor (PR)						
Negative	317 (34)	**623(66)**	** < 0.001**	63 (23)	**208 (77)**	** < 0.001**
Positive	673 (65)	367 (35)		349 (64)	197 (36)	
Human epidermal growth factor receptor 2 (HER2)						
Negative	945 (55)	788 (45)	** < 0.001**	302 (53)	265 (47)	** < 0.001**
Positive	45 (18)	**202 (82)**		50 (38)	**83 (62)**	
Epithelial growth factor receptor (EFGR)						
Negative	504 (51)	486 (49)	0.418	209 (49)	218 (51)	0.494
Positive	486 (49)	504 (51)		219 (51)	208 (49)	
Molecular subtypes						
Luminal A	614 (85)	110 (15)	** < 0.001**	315 (78)	90 (22)	** < 0.001**
Luminal B	130 (27)	358 (73)		23 (17)	118 (83)	
HER-enriched	45 (19)	195 (81)		9 (16)	47 (84)	
Basal like	37 (11)	**292 (89)**		7 (5)	**126 (95)**	
Normal like	164 (82)	35 (18)		24 (80)	6 (20)	

*P* values in bold means statistically significant

### *UBE2C* mRNA expression and related biomarkers

In the METABRIC cohort, high *UBE2C* mRNA expression showed an association with epithelial-mesenchymal transition (EMT) phenotype, specifically negative correlation with *CDH1* and positive association with *CDH2* (*p* < 0.001) (Table 2). High *UBE2C* mRNA expression also showed a strong positive association with several members of the *MMPs* family (MMP7, MMP9, MMP12, MMP14, MMP15, MMP20, MMP21 and MMP25), proliferation-related genes (CDK1, CDK2, CDK4, CDK5, CDK6, CDKN2A, CCNB1, CCNE1, CCNE2, CCNA1, CCNA2, CCNB2 and CCND3) and cell cycle-related genes (CDCA5 and CDC20) in both METABRIC and TCGA datasets (all *p* < 0.05; Table 2).

**Table 2:** Correlation of *UBE2C* mRNA expression with mRNA expression of Adhesion molecules and MMPs genes in the Molecular Taxonomy of Breast Cancer International Consortium (METABRIC) (*n* = 1980) and in the Cancer Genome Atlas (TCGA) (*n* = 854) breast cancer series

Gene names	METABRIC cohort		TCGA cohort	
	Correlation value	*p* value	Correlation value	*p* value
Adhesion molecule genes				
*CDH1*	− 0.093	** < 0.001**	− 0.020	0.553
*CDH2*	0.118	** < 0.001**	0.046	0.179
MMPs-related genes				
*MMP7*	0.114	** < 0.001**	0.253	** < 0.001**
*MMP9*	0.297	** < 0.001**	0.152	** < 0.001**
*MMP12*	0.303	** < 0.001**	0.209	** < 0.001**
*MMP14*	0.080	** < 0.001**	0.068	**0.048**
*MMP15*	0.277	** < 0.001**	0.190	** < 0.001**
*MMP20*	0.137	** < 0.001**	0.257	** < 0.001**
*MMP21*	0.041	**0.040**	0.135	** < 0.001**
*MMP25*	0.150	** < 0.001**	0.119	**0.001**
Cell cycle-related genes				
*CDK1*	0.722	** < 0.001**	0.507	** < 0.001**
*CDK2*	0.532	** < 0.001**	0.392	** < 0.001**
*CDK4*	0.400	** < 0.001**	0.278	** < 0.001**
*CDK5*	0.249	** < 0.001**	0.218	** < 0.001**
*CDK6*	0.126	** < 0.001**	0.233	** < 0.001**
*CDKN2A*	0.347	** < 0.001**	0.500	** < 0.001**
*CCNB1*	0.687	** < 0.001**	0.658	** < 0.001**
*CCNE1*	0.673	** < 0.001**	0.461	** < 0.001**
*CCNA1*	*0.220*	** < 0.001**	0.116	**0.001**
*CCNA2*	*0.819*	** < 0.001**	0.584	** < 0.001**
*CCNB2*	*0.879*	** < 0.001**	0.704	** < 0.001**
*CCND3*	0.072	**0.001**	0.117	**0.001**
*CCNE2*	0.661	** < 0.001**	0.323	** < 0.001**
*CDCA5*	0.881	** < 0.001**	0.580	** < 0.001**
*MYC*	0.085	** < 0.001**	0.281	** < 0.001**
*CDC20*	0.861	** < 0.001**	0.720	** < 0.001**

*P* values in bold means statistically significant

### *UBE2C* mRNA expression and patients’ outcome

**Fig. 1:**
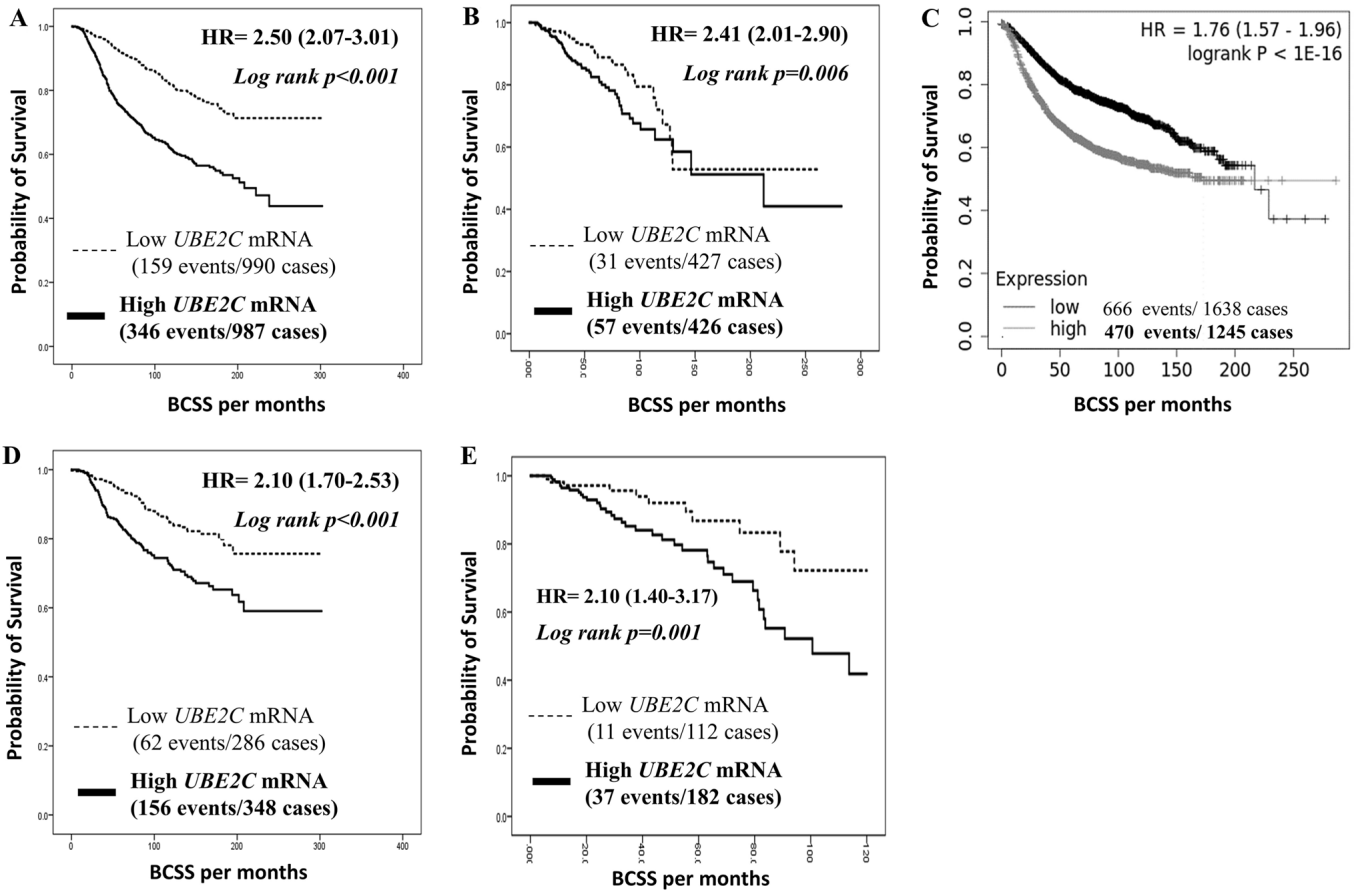
Patients’ outcomes of Breast cancer survival on Transcriptomic level. **A** Cumulative breast cancer-specific survival (BCSS) of patients stratified by *UBE2C* mRNA expression in METABRIC, **B** Cumulative BCSS of patients stratified by *UBE2C* mRNA expression in TCGA, **C** Cumulative BCSS of patients stratified by *UBE2C* mRNA expression in the KM-Plotter cohort, **D** Cumulative BCSS stratified by *UBE2C* mRNA expression in LVI-positive tumours in METABRIC, **E** Cumulative BCSS stratified by *UBE2C* mRNA expression in LVI-positive tumours in TCGA

High *UBE2C* mRNA expression was significantly associated with shorter BC specific survival (BCSS) in the METABRIC cohort (*p* < 0.001, HR = 2.50, 95% CI 2.07–3.01; Fig. 1A), in the TCGA cohort (*p* = 0.006, HR = 2.41, 95% CI 2.01–2.90; Fig. 1B) and in the KM-Plotter BC online datasets (*p* < 0.001, HR = 1.76, 95% CI 1.57–1.96; Fig. 1C). Multivariate analysis in METABRIC cohort observed that *UBE2C* expression was an independent prognostic marker significantly associated with poor patient outcome in terms of BCSS (*p* < 0.001, HR 1.90, 95% CI; 1.50–2.38), regardless of LVI, tumour size, ER and HER2 status (Table 3).

**Table 3:** Multivariate Cox proportional hazard regression analysis for predictors of breast cancer-specific survival (BCSS) in the METABRIC, TCGA and Nottingham BC cohort

Factors	Breast cancer-specific survival (BCSS) in METABRIC			Breast cancer-specific survival (BCSS) in TCGA			Breast cancer-specific survival (BCSS) in Nottingham BC cohort		
	Hazard ratio	95% CI	*p* value	Hazard ratio	95% CI	*p* value	Hazard ratio	95% CI	*p* value
UBE2C protein expression	1.9	1.50–2.38	** < 0.001**	1.22	0.69–2.14	0.502	1.6	1.10–2.30	**0.013**
Tumour size	1.87	1.53–2.30	** < 0.001**	1.24	0.68–2.30	0.483	1.34	0.93–5.64	0.113
Lymophvascular invasion (LVI)	1.64	1.33–2.04	** < 0.001**	1.71	1.01–2.90	**0.046**	2.26	1.61–3.17	** < 0.001**
Oestrogen (ER) status	0.74	0.58–0.93	**0.009**	0.64	0.36–1.17	0.147	2.3	0.93–5.64	0.072
Human epidermal growth factor receptor 2 (HER2) status	1.55	1.20–2.02	**0.001**	1.32	0.71–2.47	0.384	2.6	1.61–4.10	** < 0.001**

Significant correlations are in bold

Categorisation of the transcriptomic cohorts based on the LVI status showed that high *UBE2C* mRNA expression was strongly associated with poor patient outcome in the LVI-positive BC in both the METABRIC cohort (*p* < 0.001, HR = 2.10, 95% CI; 1.70–2.53; Fig. 1D) and the TCGA cohort (*p* = 0.001, HR = 2.10, 95% CI; 1.40–3.17; Fig. 1E). Furthermore, high *UBE2C* mRNA expression showed a non-significant association with the LVI-negative BC in the METABRIC cohort (*p* = 0.221, HR = 1.43, 95% CI; 0.80–2.60; Supplementary Fig. 2A) and the TCGA cohort (*p* = 0.537, HR 1.21, 95% CI; 0.65–2.26; Supplementary Fig. 2B).

### UBE2C protein expression

**Fig. 2:**
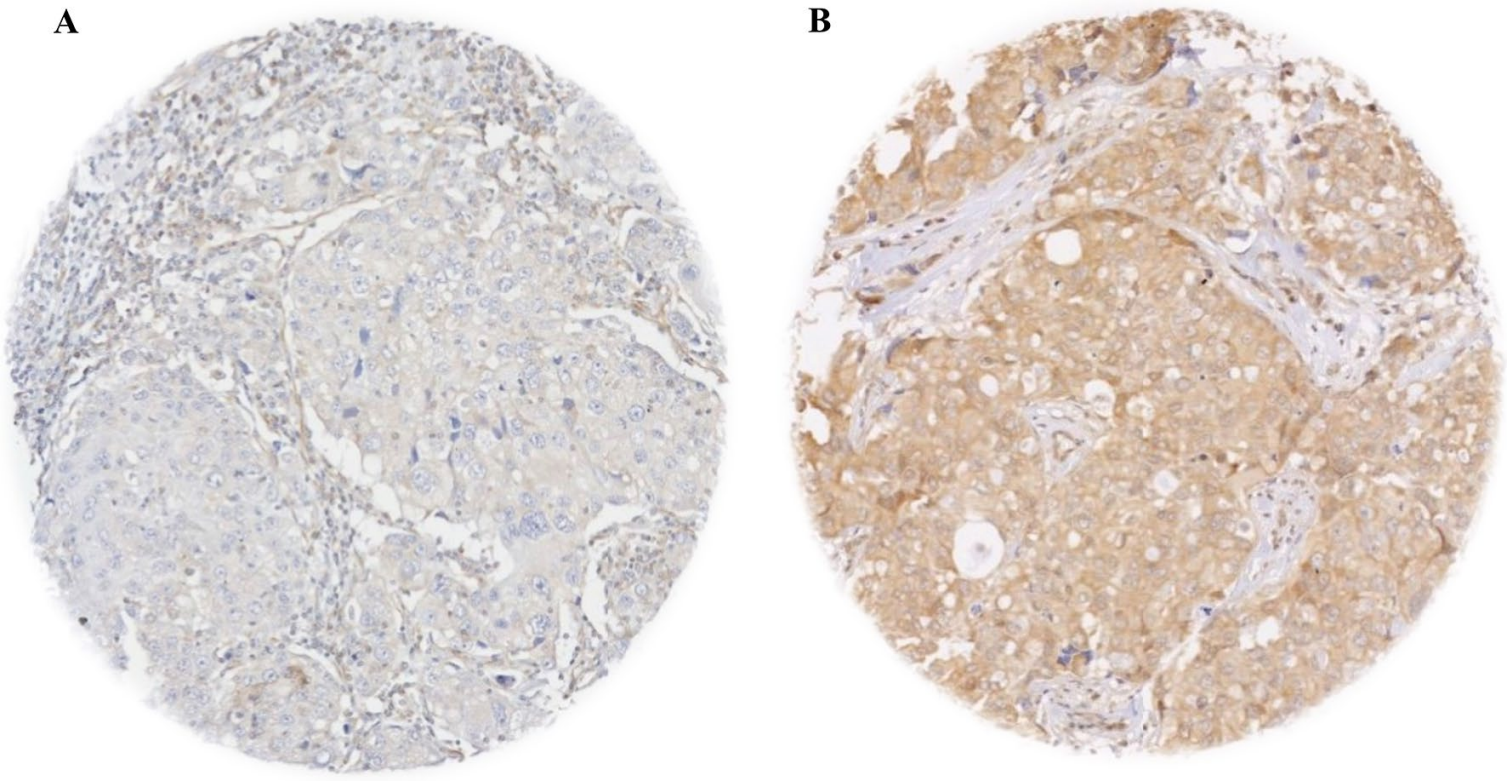
UBE2C TMAs core protein expression. **A** UBE2C weak IHC expression. **B** UBE2C strong IHC expression in invasive breast cancer TMA cores

Full-face sections of BC showed even distribution for UBE2C protein expression, which indicated the suitability of TMA to assess UBE2C protein expression. UBE2C protein expression was detected prominently in the cytoplasm of invasive tumour cells. Following double scoring of cases, a good concordance rate was obtained between the two the observers (ICC = 0.7, *p* = 0.024). Therefore, the main observer (YA) scoring was considered in the final analysis. The distribution of UBE2C protein expression showed a range from absent to high (H-score 0–160), and for dichotomisation into negative/low and high expression, the median H-score 20 was used. 376 (61%) of cases showed low expression, whereas 243 (39%) cases with high expression (Fig. 2B, C).

High expression of UBE2C was significantly associated with the presence of LVI (*p* = 0.009), and other variables of poor prognosis including the presence of nodal status, high tumour grading, larger tumour size, poor NPI, lack of ER and PR receptors expression, and HER2 positivity (Table 4). When we stratified the protein expression based on BC histological subtypes, high UBE2C protein expression was strongly associated with ductal NST BC tumour compared to other types (*p* < 0.001; Table 4).

**Table 4:** Association between UBE2C protein expression and clinicopathological characteristics of the Nottingham breast cancer cohort (*n* = 619)

Parameters	UBE2C protein expression		
	Low	High	*p* value
	*N* (%)	*N* (%)	
Tumour size			
≤ 2.0 cm	192 (66)	99 (34)	**0.011**
> 2.0 cm	180 (56)	**142 (44)**	
Lymph node status			
Negative	220 (62)	137 (38)	**0.019**
Positive	104 (41)	**153 (59)**	
Lymphovascular invasion (LVI)			
Negative	224 (68)	108 (32)	**0.009**
Positive	107 (56)	**84 (44)**	
Histological grade			
Grade 1	65 (84)	12 (16)	** < 0.001**
Grade 2	148 (74)	53 (26)	
Grade 3	163 (48)	**242 (52)**	
Histological tumour subtypes			
Ductal NST	137 (43)	**73 (33)**	** < 0.001**
Lobular	100 (31)	29 (13)	
Medullary	47 (15)	67 (30)	
Special type	37 (11)	54 (24)	
Nottingham prognostic index			
Good prognostic group	124 (79)	33 (21)	** < 0.001**
Moderate prognostic group	185 (54)	155 (46)	
Poor prognostic group	63 (54)	**53 (46)**	
Age			
< 50	133 (63)	101 (43)	0.125
> 50	239 (63)	140 (37)	
Oestrogen receptor (ER)			
Negative	61 (38)	**99 (62)**	** < 0.001**
Positive	313 (69)	143 (31)	
Progesterone receptor (PR)			
Negative	117 (47)	**132 (53)**	** < 0.001**
Positive	246 (70)	105 (30)	
Human epidermal growth factor receptor 2 (HER2)			
Negative	326 (64)	183 (36)	** < 0.001**
Positive	37 (41)	**54 (59)**	
P53			
Negative	283 (77)	137 (23)	** < 0.001**
Positive	81 (44)	**101 (56)**	
Ki67			
Negative	139 (74)	48 (26)	** < 0.001**
Positive	165 (53)	**145 (47)**	
E-Cadherin			
Negative	139 (64)	78 (36)	0.243
Positive	228 (59)	157 (41)	
N-Cadherin			
Negative	82 (66)	42 (34)	**0.033**
Positive	199 (56)	**155 (44)**	
Cyclin B1			
Negative	90 (60)	60 (40)	**0.041**
Positive	47 (41)	**67 (59)**	
Basal phenotype			
Negative	294 (64)	167 (36)	**0.002**
Positive	68 (49)	**71 (51)**	
Epithelial growth factor receptor (EGFR)			
Negative	300 (63)	171 (36)	**0.003**
Positive	66 (49)	**68 (51)**	
CDCA5			
Negative	191 (69)	84 (31)	**0.005**
Positive	109 (45)	**135 (55)**	
PI3K			
Negative	80 (71)	33 (29)	**0.019**
Positive	217 (58)	**154 (42)**	
IHC subtypes			
Luminal A	137 (65)	73 (35)	** < 0.001**
Luminal B	100 (77)	29 (23)	
HER2 enriched	37 (41)	**54 (59)**	
Triple-negative breast cancer (TNBC)	47 (42)	**67 (58)**	

*P* values in bold means statistically significant

High UBE2C protein expression was strongly correlated with high p53 expression (*p* < 0.001), high Ki67 index (*p* = 0.008), basal-phenotype biomarkers (*p* = 0.002), EGFR (*p* = 0.003), N-cadherin (*p* = 0.033), stromal immune markers CD8 and CD68 (all: *p* < 0.001), cyclin B (*p* = 0.041), and high level of PI3K (*p* = 0.019; Table 4). Among BC IHC subtypes, high UBE2C protein was indicated to be obtained more with HER2-enriched and TNBC subtype compared to other subtypes (*p* < 0.001; Table 4).

**Fig. 3:**
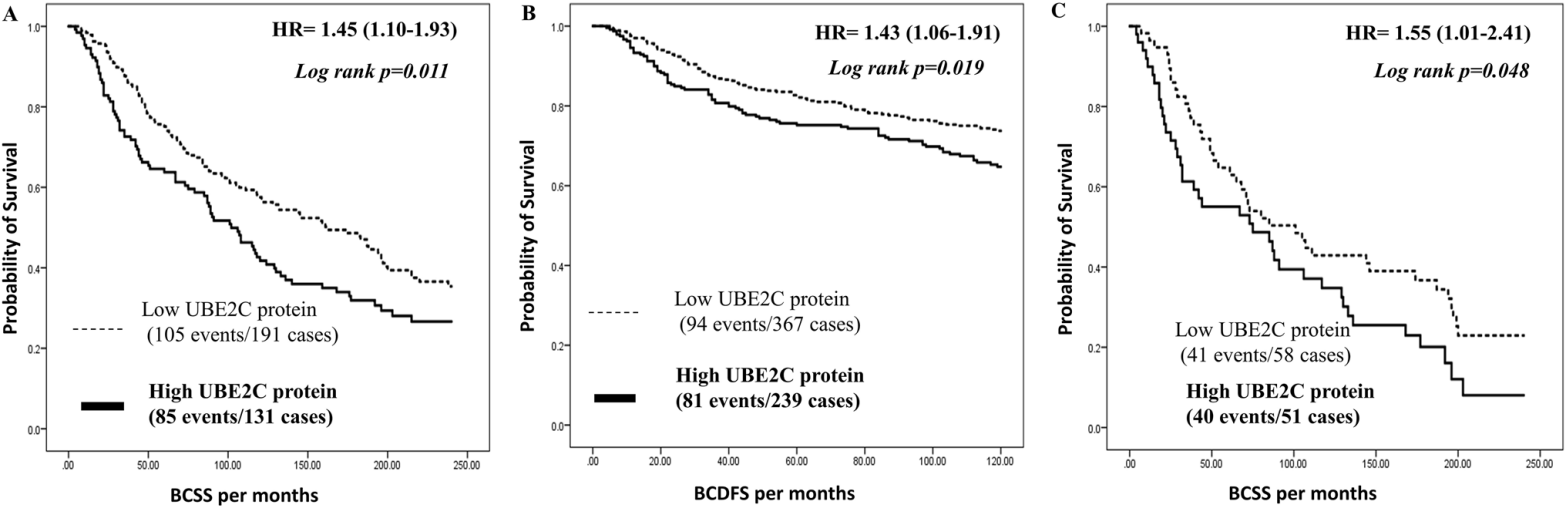
Patients’ outcomes of Breast cancer survival on UBE2C protein expression in the Nottingham cohort. **A** Cumulative breast cancer-specific survival (BCSS) of patients stratified by UBE2C protein expression. **B** Cumulative breast cancer disease-free survival (BCDFS) of patients stratified by UBE2C protein expression. **C** Cumulative BCSS stratified by UBE2C protein expression in the Nottingham LVI-positive cohort

Patients who had high UBE2C protein expression displayed poor BCSS (*p* = 0.011, HR = 1.45, 95% CI; 1.10–1.93; Fig. 3A) compared to patients who had low expression. Moreover, patients with high UBE2C protein expression showed a significant poor 10 years BC disease-free survival (BCDFS) (*p* = 0.019, HR = 1.43, 95% CI; 1.06–1.91; Fig. 3B). Multivariate analysis revealed that UBE2C expression associated with poor patients’ outcome in term of BCSS (*p* = 0.013, HR = 1.60, 95% CI; 1.10–2.30), independent on other prognostic parameters including LVI, tumour size, ER and HER2 status (Table 3).

High UBE2C protein expression was associated with worse BCSS in the LVI-positive subgroup (*p* = 0.048, HR = 1.55, 95% CI; 1.01–2.41; Fig. 3C) but not in the in the LVI-negative subgroup (*p* = 0.526, HR = 1.81, 95% CI; 0.70–2.00; Supplementary Fig. 2C).

## Discussion

BC is the most common malignancy affecting women worldwide [29]. LVI is a serious consequence in BC that contributes to cancer metastasis and hence shorter survival [8, 9]. Despite the ability of LVI to serve as a prognostic factor in BC, the underlying mechanisms and the key molecular factors involved in BC-LVI remain unknown. UBE2C is a member of the ubiquitin-conjugating enzyme family that plays a critical role in the ubiquitin–proteasome proteolytic (UPP) pathway. Dysregulation of the UPP pathway enhances tumour oncogenes and can affect tumour suppressor proteins degradation, thereby resulting in the abnormal aggregation of those proteins in the body. Accordingly, the UPP system plays a pivotal role in cancer initiation and progression [30]. Despite the recognised importance of UBE2C in relation to cancer progression, the role played by UBE2C in BC and BC-LVI remains ill defined.

Our study identified significant associations between high UBE2C expression and aggressive tumour characteristics, including larger tumour size, high tumour grade, lymph node positivity, NPI poor prognostic groups, LVI positivity, hormone receptor (ER and PR) negativity, high expression of the proliferative marker Ki67, p53 and HER2 positivity, and the HER2-enriched intrinsic BC subtype in addition to poor patient outcomes. These results are consistent with the results of previous studies that demonstrated that UBE2C is a key factor in cancer progression and prognosis [13, 14, 31]. For instance, Chao-hua Mo et al. investigated the prognostic significance of UBE2C expression at both transcriptomic level (1006 cases) and protein levels (209 BC tissue samples), and reported that high UBE2C expression is associated with worse outcome as well as aggressive tumour characteristics in BC [14]. High UBE2C protein expression was determined to exhibit a positive correlation with only HER2 at both the transcriptomic and proteomic levels when compared with the steroid receptors, which may suggest a correlation between UBE2C and HER2-enriched tumours when compared with the other molecular BC subtypes. The HER2-enriched BC type is considered one of the most aggressive types of BC, and it is significantly correlated with cancer cell adhesion [32, 33].

The positive correlation identified in this study between UBE2C expression and the presence of both LVI and nodal status implicates UBE2C in cancer invasiveness via enhancing the EMT process, which is in accordance with a previous in vivo and in vitro study of UBE2C in non-small-cell lung cancer [31]. In endometrial cancer, the silencing of *UBE2C* plays an essential role in regulating cancer cell proliferation, migration and invasion, as well as an EMT by increasing the p53 ubiquitination and stimulating its degradation activity, thereby activating cell apoptosis and preventing carcinogenesis [7]. In addition, the dysregulation in the N-cadherin levels could stimulate MMPs production and activation to provide a proper EMT, which could lead to the enhancement of tumour cell migratory behaviour and the degradation of the primary site stroma to facilitate the cancer migration process [8]. We also found high UBE2C expression to be positively associated with a high level of EGFR, which also plays a pivotal role in BC cell migration, adhesion and invasion [34]. Moreover, high UBE2C expression might contribute to the cell adhesion process via stimulating the migration of BC tumour cells through the lymphatic vessels and starting the invasion process by activating the Wnt and PI3K signalling pathway [35]. Taken together, UBE2C could act as an essential prerequisite for BC progression that is responsible for silencing the level of E-cadherin and enhancing the levels of N-cadherin and EGFR. This may result in the activation of cancer cell migration and invasion, which may explain the vital role of UBE2C in LVI and metastasis in BC.

Moreover, the mitosis-promoting factor (MPF) is an essential regulator of mitosis, which is known as an essential prerequisite for the G2/M transition [36]. In most eukaryotes, mitosis requires unique complex criteria to be activated, including formal formation, activation and cellular translocation [37, 38]. Thus, an imbalance of this complex may lead to a blockage of the mitosis process and, therefore, G2/M transition. At the transcriptomic level, the high expression of *UBE2C* exhibited significant positive associations with cyclin-related genes, which play a crucial role in both the cell cycle process (G1/S and G2/M) and cell proliferation [39]. Similar to the *UBE2C* transcriptomic level results, high UBE2C protein expression showed a significant positive correlation with cyclin B1 [40]. This positive correlation may indicate the critical role of UBE2C as a tumour oncogene during the cell cycle through enhancing the G1/S and G2/M transitions that prevent cancer apoptosis and promotes tumour cell proliferation via controlling the PI3K/AKT/mTOR signalling pathway [41]. In light of all this, the loss of UBE2C can lead to the blockage of the G2/M transition via downregulating the expression of CDK1 and cyclin B1 [42]. Similar results were obtained in melanoma; downregulation of UBE2C acts as a cell growth regulator via blocking ERK/Akt signalling pathways, and preventing the G2/M transition by activating MPF and stimulating apoptosis [42]. It was also demonstrated that UBE2C plays a pivotal role in the regulation and activation of the mTOR/PI3K/AKT pathway in cervical cancer [41]. These findings support that high UBE2C expression correlates with BC progression and invasion cascades.

This study also suggests a new avenue for exploring the therapeutic role of UBE2C as an independent biomarker that could be used to target invasive BC both directly and indirectly. Targeted anti-UBC2C therapies that block UBE2C pathways would stop and/or reduce its consequent biological actions including cellular proliferation and invasiveness. In addition, therapeutic agents targeting UBE2C would synergise the effect of other therapies including chemotherapy, anti-oestrogen medications and radiation. Previous studies showed that overexpression of UBE2C reduces the therapeutic potency of letrozole, tamoxifen, doxorubicin and leads to radio-resistance in various BC cell lines [37, 38, 43]. These findings highlight the importance of further investigating the therapeutic and predictive potential of UBE2C expression in BC.

Although this study has presented promising findings based on evidence at both the transcriptomic and proteomic levels indicating the potentially critical role of UBE2C in BC-LVI, it is important to acknowledge that it has some limitations. First, this study was based on retrospectively collected cohort data. A well-characterised randomised clinical assessment involving more cases and uniform treatment is required for the independent evaluation of UBE2C expression in BC. Second, further in vivo and in vitro functional studies are required to discover the exact molecular mechanism(s) associated with UBE2C in order to validate its potential as a prognostic marker of BC-LVI.

In conclusion, high UBE2C expression in BC is associated with both LVI positivity and poor prognostic factors. It is an independent prognostic biomarker of poor patient survival. UBE2C may play an essential role in tumour cell proliferation, migration, invasion, and metastasis. Further in vivo and in vitro functional studies are required to investigate the molecular mechanisms of UBE2C in BC and its therapeutic potential.

## Supplementary Information

Below is the link to the electronic supplementary material.

Supplementary file1 (JPG 1520 KB)

Supplementary file2 (JPG 1238 KB)

## Data Availability

The authors confirm the data that have been used in this work are available on reasonable request.
