# Supplementary figures and images for: Ubiquitin-conjugating enzyme 2C (UBE2C) is a poor prognostic biomarker in invasive breast cancer
Source: Breast Cancer Res Treat. 2022 Feb 6;192(3):529–39. doi: 10.1007/s10549-022-06531-5 (PMC8960565; doi:10.1007/s10549-022-06531-5)

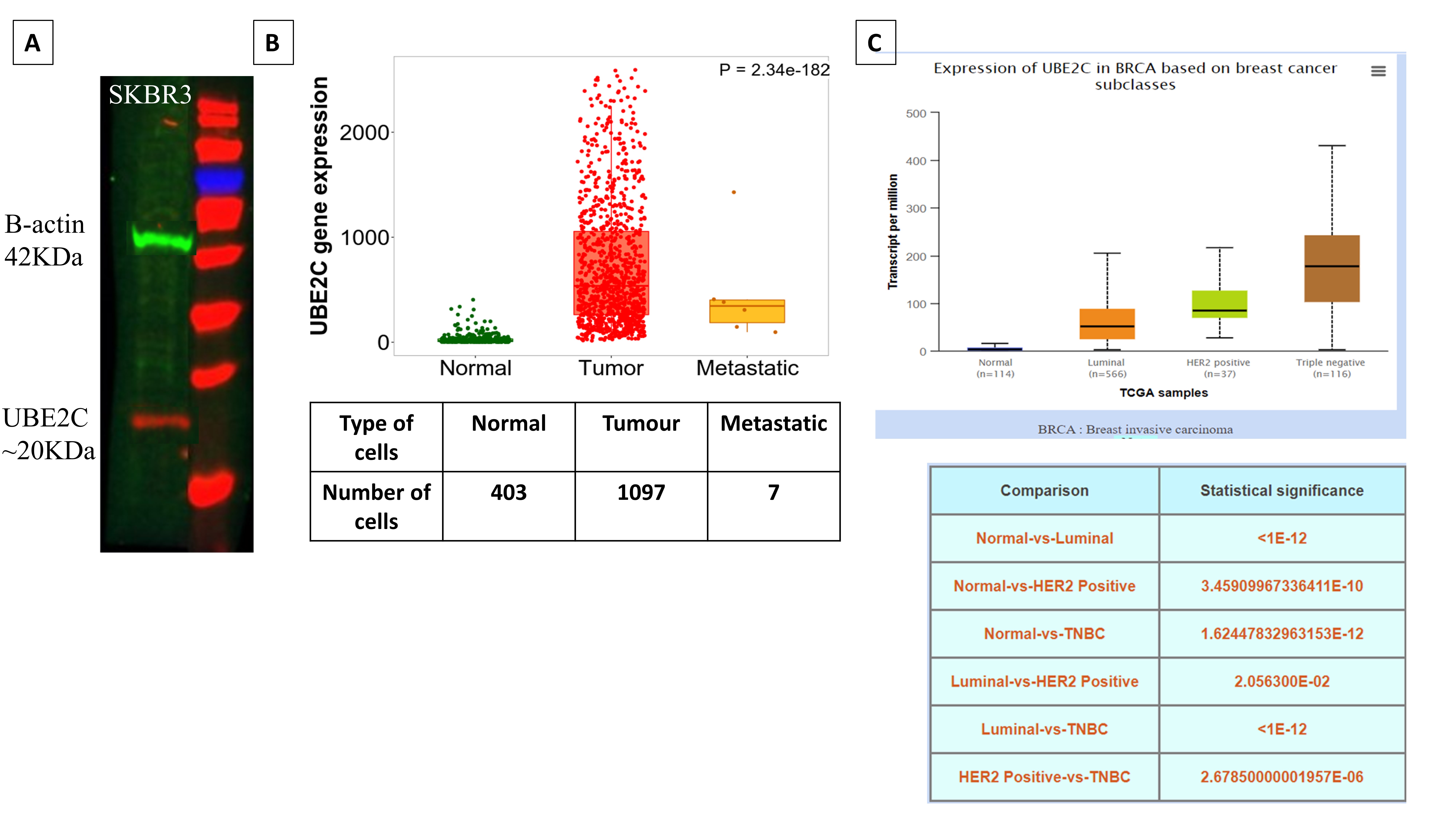

Supplement: Supplementary file 1 — Supplementary file1 (JPG 1520 KB) [file 10549_2022_6531_MOESM1_ESM.jpg]

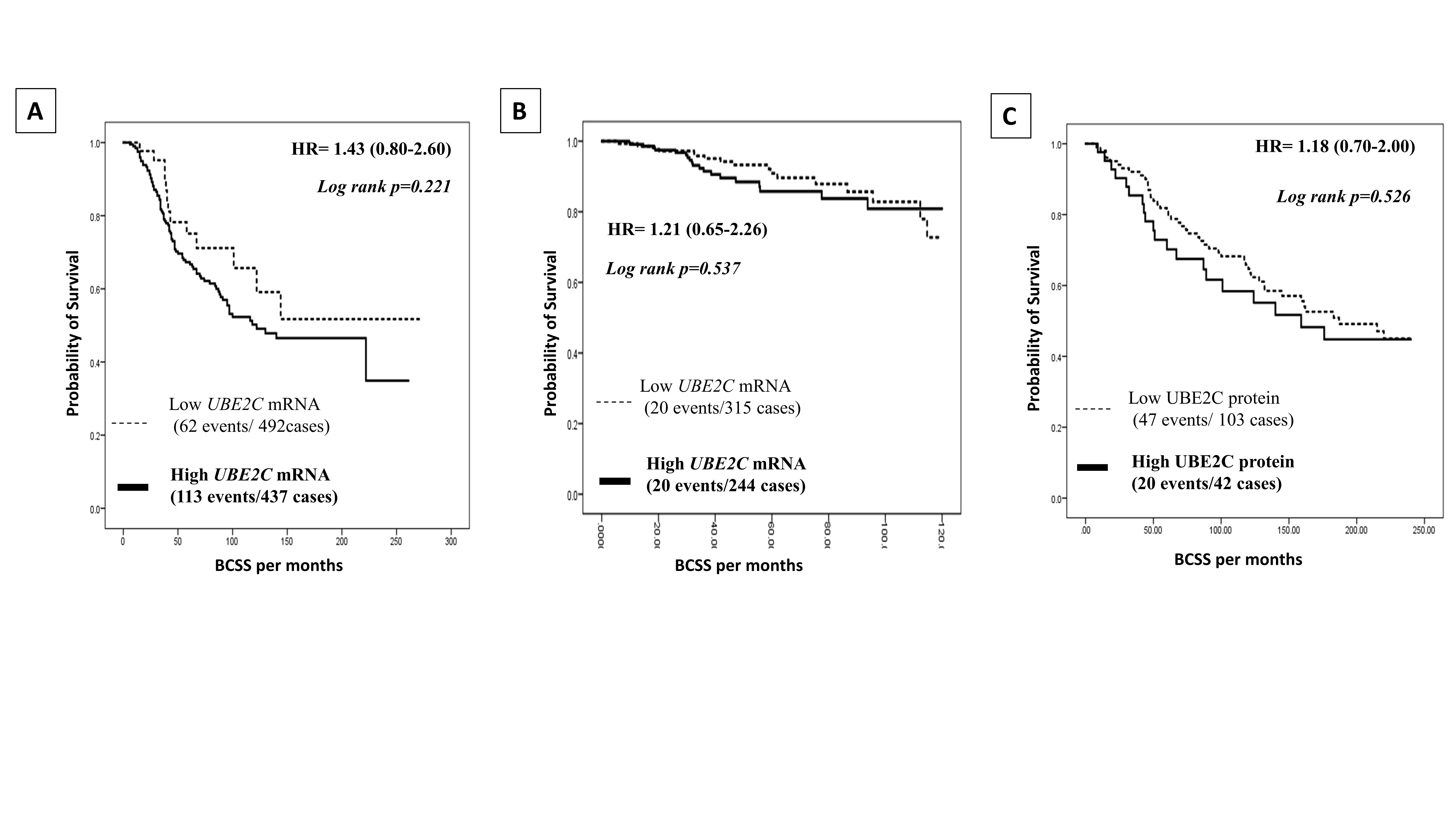

Supplement: Supplementary file 2 — Supplementary file2 (JPG 1238 KB) [file 10549_2022_6531_MOESM2_ESM.jpg]
